# Single nucleotide polymorphisms in thymic stromal lymphopoietin gene are not associated with allergic rhinitis susceptibility in Chinese subjects

**DOI:** 10.1186/1471-2350-13-79

**Published:** 2012-09-13

**Authors:** Yuan Zhang, Xiaohong Song, Yanming Zhao, Luo Zhang, Claus Bachert

**Affiliations:** 1 Department of Otolaryngology, Head and Neck Surgery, Beijing Tongren Hospital, Capital Medical University, Beijing, 100730, PR China; 2 Key Laboratory of Otolaryngology, Head and Neck Surgery (Ministry of Education of China), Beijing Institute of Otorhinolaryngology, Beijing, 100005, PR China; 3 Upper Airways Research Laboratory, Department of Oto-Rhino-Laryngology, Ghent University Hospital, De Pintelaan 185, Ghent, 9000, Belgium; 4 Beijing Institute of Otolaryngology, No. 17, HouGouHuTong, DongCheng District, Beijing, 100005, China

**Keywords:** Allergic rhinitis, Chinese subjects, Genotyping, Thymic stromal lymphopoietin, Single nucleotide polymorphism

## Abstract

**Background:**

Thymic stromal lymphopoietin (TSLP) is an epithelial cell-derived cytokine, implicated in the development and progression of allergic diseases. Recent studies have demonstrated significantly increased expression and synthesis of TSLPin nasal mucosa of patients with allergic rhinitis (AR), compared with nonallergic control subjects. Also, there is significant correlation between the level of TSLP mRNA and symptom severity in AR patients. In this study, we investigated whether polymorphisms in the TSLP gene were associated with increased risk of AR in the Chinese population.

**Methods:**

In a candidate gene association study, we tested 11 single nucleotide polymorphisms (SNPs) in the TSLP gene in 368 AR and 325 control adult Han Chinese subjects from Beijing. The 11 SNPs were selected from the Chinese HapMap genotyping dataset to ensure complete genetic coverage. AR was established by questionnaire and clinical examination, and blood was drawn from all subjects for DNA extraction. The PLINK software package was used to perform statistical testing.

**Results:**

In the single-locus analysis of AR risk, no significant differences in allele and genotype frequencies were found between AR and control subjects. Further logistic regression analyses adjusted for age and gender also failed to reveal significant associations between AR and the selected SNPs. Similarly, analysis stratified by gender, and haplotype or diplotype did not reveal any association with AR risk.

**Conclusion:**

Although TSLP presents itself as a good candidate for contributing to allergy, this study failed to find an association between specific SNPs in the TSLP gene and AR susceptibility in the Han Chinese population.

## Background

Allergic rhinitis (AR) is an inflammatory disease of the nasal mucosa induced by an immunoglobulin E (IgE)-mediated reaction in allergen-sensitized subjects. AR has increased in prevalence over the last decade, in particularly the industrialized nations, and currently affects up to 40% of the population worldwide [1]. Recent data from mainland China indicates that the prevalence of self-reported AR in major cities across China is high and ranges between 8.7%-24.1%; with around 25% of all patients suffering from persistent symptoms [2].

There is a plethora of evidence, which suggests that the airway epithelial cells are likely to play an important role in the aetiology of AR, particularly as they represent the first line of defense against inhaled microbes and foreign antigens and are capable of initiating and controlling immune responses by influencing the expression, synthesis and release of a variety of mediators that play a critical role in shaping and driving allergic inflammatory responses [3,4]. In this regard, thymic stromal lymphopoietin (TSLP) is an important epithelial cell-derived cytokine, which is expressed in skin, gut, lungs, and thymus [5], and has been referred to as a “master switch” of allergic inflammation at the epithelial cell and dendritic cell interface [6]. Indeed, several studies in humans and mouse models have implicated TSLP in the development and progression of allergic diseases, including atopic dermatitis [7-10], asthma [11-13] and AR [14-16]. Moreover, recent studies in Chinese subjects have demonstrated that the expression of TSLPmRNA and/or TSLP protein was significantly increased in the nasal mucosa/epithelia cells of patients with AR compared with the nonallergic control subjects [14,17], and there was significant correlation between the level of TSLP mRNA and symptom severity in AR patients [17].

In view of this evidence, we hypothesized that the TSLP gene is a strong candidate gene, which may influence an individual’s risk to develop AR. The aim of this study was therefore to examine whether polymorphisms in the TSLP gene are associated with an individual’s susceptibility to develop AR in a Han Chinese cohort.

## Methods

A population-based case–control association study design was used to assess the risk of AR conferred by SNPs in TSLP gene regions.

### Study subjects

Three hundred and sixty-eight adult subjects suffering from AR were recruited from the outpatient clinic of Otolaryngology, Head and Neck Surgery Department at Beijing Tongren Hospital, during February 2010 to November 2010.

All subjects had a history of AR for at least 1 year and fulfilled all of the Allergic Rhinitis and its Impact on Asthma (ARIA) guidelines [18] criteria for AR; including i) presence of persistent or discontinuous symptoms of anterior rhinorrhea, continuous sneezing, nasal obstruction and itching, ii) demonstration of a pale and edematous nasal mucosa, nasal discharge and swollen inferior turbinates by nasal endoscopy, and iii) positive skin prick test (SPT) to a panel of common allergens as shown below (Allergopharma, Reinbeck, Germany) and/or positive serum antigen-specific IgE, measured by the ImmunoCAP 100 system (Pharmacia, Uppsala, Sweden). A diagnosis of AR was further confirmed by the presence of symptoms induced by exposure to an allergen shown to produce a strong positive skin test response.

The tested antigens included house dust mite (HDM) (Der f and Der p); seasonal grass pollens (Giant Ragweed; Mugwort; Lamb’s quarers; Humulus; Chenopodium album); animal hair (especially dog and cat); molds (indoor and outdoor mustiness or floricultural environment) and cockroach. A positive SPT result was defined as a wheal greater than or equal to one half of the diameter of the histamine control and at least 3 mm larger than the diameter of the negative control [19]. Subjects were also considered to be sensitized to allergens when the serum IgE was ≥0.35 kU/l.

AR subjects with i) co-morbid asthma, eczema, or any other allergic disease; ii) hypertension, diabetes or other chronic diseases; or iii) tumor in the nasal cavity or any other inflammatory nasal disease were excluded. The diagnosis of asthma was confirmed by a chest physician according to Global Initiative for Asthma (GINA) guidelines [20].

A total of 325 adult healthy control volunteers were also recruited from an ethnically similar local population to determine background population allele frequencies. None of these subjects had a history of allergic or any nasal disease, nor demonstrated any abnormal clinical features in the nasal cavity, or a positive SPT to any of the common allergens as shown above.

All subjects were ethnic Han Chinese, who had lived in the Beijing region, China, over long periods and provided written informed consent prior to entry in the study. The study protocol was approved by the Ethics Committee of Beijing Tongren Hospital and performed in accordance with the guidelines of the World Medical Association’s Declaration of Helsinki.

### Selection of polymorphisms in the human TSLP gene

The International Haplotype Mapping (HapMap) (http://www.hapmap.org) SNP databases were used to select tSNPs in the TSLP gene region. The screened region extended 10 kilobases upstream of the annotated transcription start site and downstream at the end of the last exon in the gene. The tSNPs were selected to extract most genetic information in the region using the CHB genotyping data from the HapMap database (HapMap data rel 27 Phase II + III, Feb2009) [21]. Genotyping data was obtained for 25tSNPs for TSLP in this dataset and loaded in the Haploview software version 4.2 (http://www.broad.mit.edu/haploview/haploview-downloads ) [22]. Further selection of the eventual tSNPs to be investigated was made using a pair-wise tagging algorithm [22]; setting the Hardy-Weinberg p value, minor allele frequency (MAF), and r^2^ threshold values at 0.01, 0.05 and 0.8, respectively. The linkage disequilibrium (LD) pattern of the TSLP gene in the CHB population exhibited strong LD in several groups of tSNPs (r^2^ greater than or equal to 0.8), indicating that the most common SNPs could be captured by a subset of tagging SNPs [23]. Subsequently, 11TSLP gene SNPs (including rs1545169, rs764917, rs12653736, rs1837253, rs12654933, rs10455025, rs11466741, rs13156086, rs6886755, rs252706 and rs2416259) were selected to represent the entire 25 loci for genotyping.

### Single nucleotide polymorphism genotyping

DNA was isolated from peripheral blood leukocytes using the DNA Isolation Kit for Mammalian Blood (Roche, Indianapolis, USA), and stored at 4°C prior to further investigation within 2 days.Genotyping of the selected SNP was performed using iPLEX chemistry on a matrix-assisted laser desorption/ ionization time-of-flight mass spectrometer (Sequenom, San Diego, California) according to the manufacturer’s instructions [24]. The polymerase chain reaction (PCR) and extension primers were designed using MassARRAY Assay Design 4.0 software (Additional file 1: Table S1).

PCR was carried out in standard 384-well plates, using 5 μL of a reaction mix comprising 10 ng of genomic DNA, 0.5 units of Taq polymerase (HotStarTaq, Qiagen), 500 μmol of each deoxynucleotide triphosphate, and 100 nmol of each primer per well. PCR thermal cycling was carried out in an ABI-9700 instrument (GeneAmpPCR system 9700, ABI, California) for 15 min at 94°C, followed by 45 cycles of 20 s at 94°C, 30 s at 56°C, and 60 s at 72°C. At the end of cycling, 2 μL of a solution containing 0.3 units of shrimp alkaline phosphatase was added to each well and the reaction was incubated at 37°C for 20 min, and then at 85°C for 5 min to terminate the reaction.

After adjusting the concentrations of extension primers to equilibrate signal-to-noise ratios, the post-PCR primer extension reaction of the iPLEX assay was carried out in a final volume of 9 μL extension reaction mixture containing 0.2 μL of termination mix, 0.04 μL of DNA polymerase (Sequenom, Inc.), and 625 to 1,250 nmol/L extension primers. A 200-short-cycle program was used for the iPLEX reaction; involving an initial denaturation by incubation for 30 s at 94°C, followed by incubation for 5 s at 94°C, five cycles of 5 s at 52°C, and 5 s at 80°C. Forty additional annealing and extension cycles were looped back to 5 s at 94°C, five cycles of 5 s at 52°C and 5 s at 80°C, and the final extension was carried out at 72°C for 3 min. The final reaction mixture was cooled to 4°C and each sample was desalted using 6 mg of clean resin and a dimple plate. The final product was transferred to a 384-well Spectro-CHIP (Sequenom, Inc.), and analyzed in a Compact Mass Spectrometer, using the MassARRAYTyper4.0 Software. The PCR assay was arrayed with two no-template controls and four duplicated samples in each 384-well format as quality controls. All genotyping results were generated and checked by an investigator blinded to the clinical status of the subject from whom the sample was derived.

### Statistical analyses

Data were initially processed for suitability for further statistical evaluation using the Haploview version 4.2software. Hardy-Weinberg equilibrium (HWE) of each SNP was assessed in controls only and a threshold P <0.001 was regarded to indicate deviation from the HWE. The data were filtered further by additionally assessing the minor allele frequency (MAF), non-missing genotype percentage and other criteria in both the AR patients and control subjects; after setting the MAF and non-missing genotype percentage thresholds at <0.001 and <95%, respectively.

Differences in frequencies of the alleles and genotypes between the AR subjects and control subjects were evaluated using the chi-square test and HWE was tested by the chi-square test for goodness of fit according to the web-based program: http://ihg.gsf.de/cgi-bin/hw/hwa1.pl. A P-value of 0.05 was considered significant. Akaike’s information criteria (AIC) were used to select the most parsimonious genetic model for each SNP. Odds ratios (ORs) and 95% confidence intervals (CIs) were calculated by unconditional logistic regression analysis, adjusted for age and gender. Stratification analyses were also performed by variables of interest, such as gender. These analyses were conducted using the STATA statistical package (version 11.0; Stata Corp LP, College Station, TX, USA).

The pair-wise linkage disequilibrium (LD) among the SNPs was examined using Lewontin’s standardized coefficient D’ and LD coefficient r^2^[25], and haplotype blocks were defined by the method of Gabriel et al. [26] in Haploview 4.2 with default settings. The population as a whole, including AR patients and controls, were utilized in block definition. The HAPLO.STATS package in software language R developed by Schaidand colleagues [27] (http://www.mayo.edu/hsr/Sfunc.html) was used for the haplotype analysis and PHASE 2.1 Bayesian algorithm [28] was used to estimate the haplotype frequencies. Haplotypes with a frequency of less than 0.03 were pooled into a combined group and empirical P-values based on 100,000 simulations were computed for the global and individual haplotype score tests. Diplotype (haplotype dosage, an estimate of the number of copies of the haplotype) was the most probable haplotype pair for each individual. Unconditional logistic regression analyses, adjusted for age and gender, were conducted to estimate ORs and 95% CIs for participants carrying one to two copies versus zero copy of each common haplotype for the dichotomized diplotypes.

The statistical power for the study was calculated using G*Power 2 software (http://www.psycho.uni-duesseldorf.de/aap/projects/gpower/). The statistical power for comparison of AR vs. control was 95.01%, with the sample size of 368 AR patients and 325 controls; the α was 0.05 and the β was 0.2.

## Results

### Population characteristics

The demographic characteristics of the study population are shown in Table 1. Both the AR and control groups were well matched with respect to age, although the AR group consisted of more males (60.05%) than females (39.95%). In contrast there were approximately equal numbers of males and females in the control group (53.54% vs 46.46% m/f). The Pearson Chi-Square test of the ratios for male/female between the control and AR groups showed that these were not significantly different (P = 0.084). The mean total serum IgE measurements for AR and control groups were 325.35 ± 31.70 and 69.14 ± 9.35 IU/ml respectively.

**Table 1:** Demographic characteristics of the study population

**Demographic index**	**AR (n = 368)**	**Controls (n = 325)**
Age Mean (Range) (years)	27.08 ± 0.77 (18–71)	36.22 ± 0.84 (18–78)
Sex, Male/Female, No. (%)	227 (60.1) /151 (39.9)	175 (53.0) / 155 (47.0)
Total IgE Mean (Range), kU/l	325.35 ± 31.70 (6.9-5000)	69.14 ± 9.35 (2–906)

AR = Allergic rhinitis subjects.

### Individual SNP association analysis

The initial quality tests for the SNPs in the TSLP gene selected for genotyping demonstrated that two SNPs (rs1545169and rs764917) were not suitable for study, as indicated by significant deviation from the Hardy–Weinberg equilibrium (HWE) threshold of P <0.001 (Table 2). The data for these two loci were therefore excluded from further analyses, and overall a total of nine SNPs (Table 2) from chromosome 5q22.1 were chosen for further assessment. In single-locus analyses for AR risk, the allele frequencies for any of the nine selected tag SNPs were not significantly different between the AR and control subjects (P >0.05) (Table 2).

**Table 2:** SNPs genotyped for TSLPgene

**Gene: locus and OMIMNo.**^**a**^	**No.**	**SNP_ID**	**Chromosome Position**^**b**^	**Intermarker distances (bp)**	**Genic location**	**Base Change**	**MAF**^**c**^			**P**^**f**^	**P value for HWE**^**g**^**test**
							**NCBI**^**d**^	**Case**^**e**^	**Control**		
	1	rs1545169	110427275	——	5′ near gene	T/G	0.476	0.292	0.278	0.6928	**<0.0000**
	2	rs764917	110428406	1131	5′ near gene	A/C	0.205	0.284	0.289	0.5036	**0.0004**
	3	rs12653736	110428526	120	5′ near gene	G/T	0.062	0.104	0.113	0.8689	0.6270
	4	rs1837253	110429771	1245	5′ near gene	T/C	0.389	0.467	0.460	0.5430	0.7337
	5	rs12654933	110430654	883	5′ near gene	C/A	0.244	0.154	0.144	0.7575	0.8902
	6	rs10455025	110432898	2244	5′ near gene	A/C	0.067	0.050	0.048	0.9790	0.3650
TSLP: 5q22.1	7	rs11466741	110436604	3706	Intron 2	C/T	0.211	0.313	0.329	0.8127	0.2428
	8	rs13156086	110443368	6764	3′ near gene	A/C	0.144	0.239	0.251	0.6890	0.7148
	9	rs6886755	110443500	132	3′ near gene	G/T	0.089	0.077	0.073	0.9559	0.5559
	10	rs252706	110444759	1259	3′ near gene	G/A	0.337	0.327	0.337	0.8165	0.0635
	11	rs2416259	110447641	2882	3′ near gene	T/C	0.411	0.369	0.366	0.6565	0.1416

a. OMIM, Online Mendelian Inheritance in Man (http://www.ncbi.nlm.nih.gov/Omim).

b. SNP position in Chromosome 5 in the NCBIdbSNP database (http://www.ncbi.nlm.nih.gov/SNP).

c. MAF, minor allele frequency.

d. MAF for Chinese in the NCBIdb SNPs database.

e. allergic rhinitis subjects.

f. P value for difference in allele distributions between allergic rhinitis and control subjects.

g. HWE, Hardy–Weinberg equilibrium.

Similarly, the genotypes of any of the nine selected SNPs in AR and control were not found to be associated with AR susceptibility (Table 3). Moreover, age and gender adjusted logistic regression analyses revealed that in the codominant-effect model, no significant protective or risk effects against AR were associated with SNPs in TSLP gene, compared with wild-type carriers (Table 3), as assessed by the Akaike’s information criteria (AIC). In addition, stratified analyses of AR associations demonstrated no significant associations with any of the selected SNPs among males or females (data not shown).

**Table 3:** Genotype frequencies of 9 tag SNPs among AR and control subjects, and their associations with AR risk

**SNP ID**	**Genotype**	**AR subjects**		**Control subjects**		**P (2 df)**^**a**^	**Logistic regression**	
		**No.**	**Frequency**	**No.**	**Frequency**		**OR (95%CI)**	**P**^**b**^
rs12653736	GG	290	79.89%	255	78.95%	0.852	1.00 (referent)	
	GT	66	18.18%	63	19.50%		0.999 (0.667-1.496)	0.996
	TT	7	1.93%	5	1.55%		1.073 (0.322-3.572)	0.909
rs1837253	TT	98	26.85%	90	28.66%	0.828	1.00 (referent)	
	TC	186	50.96%	159	50.64%		1.043 (0.719-1.514)	0.823
	CC	81	22.19%	65	20.70%		1.078 (0.686-1.696)	0.744
rs12654933	CC	269	73.30%	237	73.37%	0.659	1.00 (referent)	
	CA	86	23.43%	79	24.46%		1.012 (0.701-1.463)	0.948
	AA	12	3.27%	7	2.17%		1.151 (0.429-3.092)	0.780
rs10455025	AA	333	90.74%	292	90.40%	0.622	1.00 (referent)	
	AC	33	8.99%	31	9.60%		0.867 (0.503-1.492)	0.605
	CC	1	0.27%	0	0.00%		NA^c^	NA^c^
rs11466741	CC	134	46.69%	112	46.67%	0.879	1.00 (referent)	
	CT	121	42.16%	98	40.83%		0.993 (0.675-1.461)	0.972
	TT	32	11.15%	30	12.50%		0.816 (0.454-1.469)	0.498
rs13156086	AA	198	58.24%	169	56.52%	0.909	1.00 (referent)	
	AC	120	35.29%	110	36.79%		0.903 (0.639-1.277)	0.564
	CC	22	6.47%	20	6.69%		0.826 (0.423-1.613)	0.576
rs6886755	GG	317	86.38%	276	85.71%	0.624	1.00 (referent)	
	GT	47	12.81%	45	13.98%		0.793 (0.501-1.256)	0.323
	TT	3	0.82%	1	0.31%		2.535 (0.255-25.235)	0.428
rs252706	GG	163	44.54%	149	46.27%	0.831	1.00 (referent)	
	GA	155	42.35%	129	40.06%		1.174 (0.837-1.646)	0.353
	AA	48	13.11%	44	13.66%		1.120 (0.684-1.832)	0.653
rs2416259	TT	139	38.08%	126	42.14%	0.335	1.00 (referent)	
	TC	176	48.22%	127	42.47%		1.361 (0.960-1.928)	0.083
	CC	50	13.70%	46	15.38%		1.122 (0.689-1.828)	0.644

AR = Allergic rhinitis.

a. Global P values [2 degrees of freedom (df)]: genotype frequencies in AR and control subjects were compared using a χ^2^ test with 2 df.

b. P values from unconditional logistic regression analyses, adjusted for age and gender.

c. NA, not available because of the rarity of genotype.

### LD Analysis and Haplotype Block Structure

**Figure 1:**
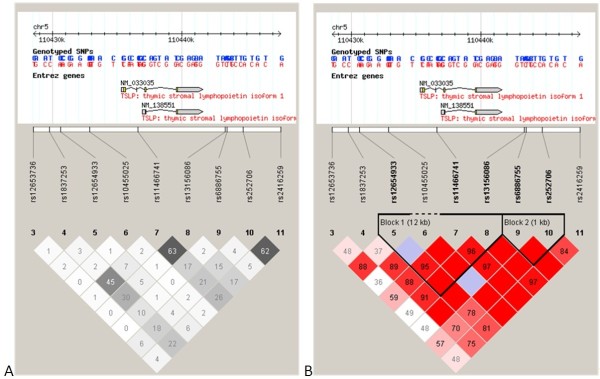
**Graphical representation of the SNP locations and LD structure of the candidate gene.** Each figure was composed of chromosome scale (the top line with even division), the transcription string (the thick bars represent exon (yellow) or UTR (blue), the thin lines represent intron), SNP scale (the hollow bar with scales representing SNPs location), and graphic of LD (black-and-white) or block definition (flammulated). The measure of LD (D’) among all possible pairs of SNPs is shown graphically according to the shade of color (**A**), where white represents very low D’ and dark represents very high D’. The numbers in squares are D’ values (D’ x 100). The measure of LD (r^2^) among all possible pairs of SNPs is shown graphically according to the shade of color (**B**), where white represents very low r^2^ and scarlet represents very high r^2^. The numbers in squares are r^2^ values (r^2^ x 100).

Figure 1 shows plots of the pair-wise LD (r^2^ and D’) values for the tag SNPs and LD structures in the selected region of chromosome 5 region (Figure 1-A). Two blocks were identified in AR vs. control subjects in the selected region in chromosome 5; of which block 1 encompassed the 5′and 3′ regions of the TSLP gene as well as the whole TSLP gene region, whereas block 2 encompassed the 3′ region of the TSLP gene (Figure 1-B).

### Haplotype analysis

Table 4 summarizes the associations between frequencies of the haplotypes in blocks r rs12654933-rs11466741-rs13156086 and rs6886755-rs252706 and the risk of AR. None of the haplotypes investigated was found to be associated with increased risk of AR in the cohort studied. Likewise, no association was found between the diplotypes in the two blocks of AR and AR risk (Table 5).

**Table 4:** Associations between common haplotypes near TSLP gene and AR risk

**Block**	**AR subjects**		**Control subjects**		**P**^**a**^	**P**_**sim**_^**b**^	**Hap. Score**^**c**^	**Logistic regression**		**Global score test**^**f**^
	**No.**	**Frequency**	**No.**	**Frequency**				**OR (95% CI)**	**P**^**d**^	
Block1: rs12654933-rs11466741-rs13156086										
CCA	382	52.10%	339	52.30%	0.9211	0.6622	0.44067	1.00 (referent)		Global-stat = 0.49867, df = 4, p-val = 0.97363, P_sim_^b^ = 0.97596
CTC	180.3	24.60%	165.1	25.50%	0.6940	0.5274	−0.63567	0.933 (0.716-1.217)	0.610	
ACA	109	14.90%	91.5	14.10%	0.7014	0.80357	0.25053	1.029 (0.743-1.426)	0.863	
CTA	56.5	7.70%	48.4	7.50%	0.8739	0.83692	−0.20715	1.002 (0.604-1.664)	0.993	
Others	5.1	0.70%	3.9	0.60%	NA^e^	NA^e^	NA^e^	NA^e^	NA^e^	
Block2: rs6886755-rs252706										
GG	430.8	58.50%	383.1	58.90%	0.8761	0.70783	−0.37559	1.00 (referent)		Global-stat = 0.71243, df = 2, p-val = 0.70032, P_sim_^b^ = 0.70097
GA	252.1	34.30%	219.2	33.70%	0.8355	0.4783	0.70725	1.073 (0.845-1.362)	0.563	
TG	53.1	7.20%	47.7	7.30%	0.9336	0.55034	−0.60092	0.913 (0.592-1.408)	0.681	

AR = Allergic rhinitis.

a. P value for difference in haplotype frequency between AR and control subjects.

b. Generated by permutation test with 100,000 times simulation.

c. A positive (or negative) score for a particular haplotype would have suggested that the haplotype was associated with increased (or decreased) risk of allergic rhinitis.

d. P values from unconditional logistic regression analyses, adjusted for age and gender.

e. NA, not available because of the rarity of genotype.

f. df, degrees of freedom.

**Table 5:** Diplotype analysis of common haplotypes near TSLP gene with AR risk

**Block**	**0-copy**		**1-copy logistic regression**			**2-copy logistic regression**			**P (2 df)**^**b**^
	**AR/C**	**OR (95%CI)**	**AR/C**	**P**^**a**^	**OR (95%CI)**	**AR/C**	**P**^**a**^	**OR (95%CI)**	
Block1: rs12654933-rs11466741-rs13156086									
CCA	85/75	1.000 (referent)	167/144	0.583	1.118 (0.750-1.667)	116/106	0.770	1.066 (0.695-1.633)	0.947
CTA	332/294	1.000 (referent)	33/30	0.709	0.902 (0.526-1.548)	3/1	0.414	2.606 (0.262-25.927)	0.676
CTC	208/178	1.000 (referent)	136/126	0.561	0.907 (0.653-1.260)	24/21	0.739	0.896 (0.468-1.713)	0.884
ACA	270/240	1.000 (referent)	86/78	0.869	1.031 (0.714-1.491)	12/7	0.763	1.164 (0.434-3.125)	0.668
Block2: rs6886755-rs252706									
GG	67/60	1.000 (referent)	171/147	0.888	1.032 (0.667-1.595)	130/118	0.796	0.942 (0.600-1.480)	0.946
GA	164/149	1.000 (referent)	156/132	0.436	1.143 (0.817-1.600)	48/44	0.678	1.110 (0.679-1.815)	0.893
TG	318/279	1.000 (referent)	47/45	0.342	0.800 (0.505-1.267)	3/1	0.423	2.558 (0.257-25.465)	0.629

AR = Allergic rhinitis subjects; C = control subjects.

a. P values from unconditional logistic regression analyses, adjusted for age and gender.

b. Global P values [2 degrees of freedom (df)]: diplotype frequencies in cases and controls were compared using a χ2 test with 2 df.

## Discussion

In this study, we aimed to evaluate the contribution of SNPs in the TSLP gene towards AR susceptibility in Han Chinese subjects by employing a population-based case–control association analysis.Our study demonstrated that the allele frequencies for none of the nine tag SNPsselected for AR risk assessment were significantly different between patients with AR and control subjects. Similarly, no differences were found between the AR patients and control subjects for either the genotype distributions of the selected SNPs or the haplotype frequencies. Overall, these findings suggest that although the TSLP gene presents itself as a good candidate involved in the development of allergy, this gene is unlikely to be associated with increased susceptibility to AR in Han Chinese subjects.

The TSLP gene is located on human chromosome 5q22, near the gene cluster encoding T helper (Th) 2 cytokines [29,30], and plays a critical role in Th2 cell differentiation [31]. Association studies of allergy related phenotypes using genetic polymorphisms have been performed in different populations [32]; with some recent studies showing possible roles of human genetic polymorphisms of the TSLP gene in allergic diseases. One study by Harada and colleagues [33] demonstrated that the SNP rs3806933 in the promoter region of TSLP created a binding site for the transcription factor activating protein (AP)-1, and in vitro enhanced AP-1 binding to the regulatory element. In a more recent study these authors demonstrated that TSLP gene promoter polymorphisms (rs3806933 and rs2289276) were significantly associated with disease susceptibility in both childhood atopic and adult asthma [33]. Similarly, Liu and colleagues [34] demonstrated that one particular variant of TSLP (rs1898671) contributed to asthma susceptibility in admixed urban populations and that the risk of asthma was significantly increased in ex-smokers; suggesting gene and environment interaction. In contrast, a large study from Canada demonstrated that variant rs1837253, which is 5.7 kb upstream of the transcription start site of the TSLP gene, was associated with protection from asthma, atopic asthma, and airway hyperresponsiveness [35]. More recently, a genome-wide association study in a Japanese population-based cohort of adult asthma utilizing fine mapping analysis of the region on chromosome 5q22 using 13 tag SNPs showed that rs1837253 represented an associated LD block spanning 88 kb that included two genes, TSLP and WDR36. The authors further concluded that TSLP was the most plausible susceptibility gene in this locus [36]. Similarly, a large consortium-based genome-wide study has demonstrated significant association between asthma and several other SNPs [37]. However, with respect to the association between TSLP polymorphisms and AR, the study by Bunyavanich and colleagues [16] indicated that TSLPSNP rs1837253 was associated with reduced odds for AR in boys with asthma. In the present study, we selected the representative tag SNPs in and near the TSLP gene region and the SNPs confirmed to be the candidate locus associated with allergy in previous studies. In the present study, rs1898671 and rs3806933 were substituted for rs10455025 and rs12110124 in terms of the r^2^value (>0.8), but despite the inclusion of these main described TSLP variants (rs1837253, rs1898671 and rs3806933) in the adult Han Chinese population, we were not able to demonstrate any significant association between these variants and AR susceptibility, similar to that shown by Bunyavanich and colleagues [16]. However, unlike the study of Bunyavanich and colleagues [16] which investigated individuals with comorbid AR and asthma, a major strength of our study is that subjects with AR and asthma were excluded and all subjects in this study only had AR. This is of particular relevance because up to 80% of asthmatics have AR and as noted from several studies described above, TSLP is a well replicated asthma susceptibility gene. Thus, it is possible that the lack of an association between the TSLP gene and AR susceptibility noted in our study is an accurate reflection of the real-life situation, because the TSLP gene is associated with asthma but not AR.

It is of interest that there is evidence which suggests that gender might modify the role of TSLP in asthma. Transgenic expression of TSLP in mice leads to perivascular leukocytic infiltration with prominent eosinophilia, with increased severity noted in female mice compared to malemice [38]. Hunninghake and colleagues [39] reported a sex-specific association between a polymorphism, rs2289276, and serum total IgE in girls in two independent populations. More recently, these authors demonstrated that TSLP polymorphisms were also associated with asthma in a sex-specific fashion [40]. The T allele of rs1837253 was significantly associated with a reduced risk of asthma in males only, whereas the T allele of rs2289276 was significantly associated with a reduced risk of asthma in females only [40]. However, in stratified analyses of AR associations with gender, no SNPs showed significant associated effects among either male nor female groups in present study.

Despite the possibility that the TSLP gene is generally not associated with risk of AR, as discussed above, it is also possible that the discordance noted between the findings of the present study and previous studies for any associations between TSLP polymorphisms and risk of AR [14,16,17], may be a consequence of difference in study protocols as well as some limitations of the present study. Firstly, we cannot completely exclude the possibility that some of our findings are false negative due to the relative small sample size.It could also be that SNPs in the TSLP gene region confer a relatively small risk of developing AR, which we could not detect. Future efforts to identify TSLPSNPs carrying a smaller TSLP risk will thus require a larger sample size than has been used here. A second consideration is the ethnic variability in SNP frequency known for the gene. It is clear that in this study, we have replicated SNPs associated in other asthmatic populations and the representative tag SNPs in terms of the Hapmap CHB population data, but have not performed extensive fine mapping studies of the gene which might identify other SNPs with increased risk. Moreover, as with many other complex disorders, AR is thought to be the result of a complicated network of numerous susceptibility loci, many of which exert additive or synergistic effects, but have only a small role when considered in isolation [41,42]. Further studies including genes involved in the Th2 pathway are needed in larger cohorts of adult Chinese subjects with AR. However, it is important to note that based on the current study, a particular TSLP variant alone does not show a significant association with the development of AR. Although estimation of TSLP protein in the nasal mucosa of the subjects would undoubtedly have provided further direct information in the elucidation of any association between a TSLP gene variant and susceptibility/development of AR in this cohort, it was unfortunately not possible to accomplish this because of the difficulty in convincing both the AR and control subjects to provide nasal mucosal samples. Moreover, it was also not possible to perform this aspect of the study from the ethical aspects.

## Conclusion

In conclusion, although TSLP presents itself as a good candidate for contributing to allergy, particularly asthma, this study failed to find an association between specific SNPs in the TSLP gene region and AR susceptibility in Han Chinese subjects. However, this finding needs to be confirmed in a larger cohort of patients with only AR.

## Competing interests

The authors declare that they have no competing interests.

## Authors’ contributions

YZ participated in the design of the study, carried out the molecular genetic studies and drafted the manuscript. XS conceived of the study and helped to draft the manuscript. YZ performed the statistical analysis. LZ participated in its design and coordination, conceived of the study and helped to draft the manuscript. CB participated in its design and helped to draft the manuscript. All authors read and approved the final manuscript.

## Funding

This work was supported by grants from the National Science Fund for Distinguished Young Scholars (81025007), National Natural Science Foundation of China (30973282 and 81100706), Beijing Science and Technology Program (Z111107055311040 and KZ200910025008) and Beijing Nova Program (2010B022).

## Pre-publication history

The pre-publication history for this paper can be accessed here:

http://www.biomedcentral.com/1471-2350/13/79/prepub

## Supplementary Material

Additional file 1 **Table S1.** Primers used in the screening of SNPs by MassArray. Click here for file
