# Supplementary material for: Single nucleotide polymorphisms in thymic stromal lymphopoietin gene are not associated with allergic rhinitis susceptibility in Chinese subjects
Source: BMC Med Genet. 2012 Sep 13;13:79. doi: 10.1186/1471-2350-13-79 (PMC3459694; doi:10.1186/1471-2350-13-79)
Supplement: Additional file 1 — Table S1. Primers used in the screening of SNPs by MassArray. [file 1471-2350-13-79-S1.doc]

**SUPPORTING INFORMATION:**

Primers used in the screening of SNPs by MassArray.

| SNP | Alleles | Position | Function | Primers | Extension Primers |
| --- | --- | --- | --- | --- | --- |
| 1545169 | T/G | 110427275 | Unknown | ACGTTGGATGCTTACAGTTCTGAATGCTGG  ACGTTGGATGGCAAAACTAAGGCCATCTGC | CTGAATGCTGGAAGTCC |
| 764917 | A/C | 110428406 | Unknown | ACGTTGGATGGAACAGTTATTCCAAAAATGC  ACGTTGGATGTCCAGGTTTTGTAAAATCGC | AAAAATGCATTTGAAAAGAAGA |
| 12653736 | G/T | 110428526 | Unknown | ACGTTGGATGGCTCTTGTTCCTCAACATTTG  ACGTTGGATGAAGACTTGGGAACTTAGGAG | CTCATAACTCATATAGAGTACCT |
| 1837253 | T/C | 110429771 | Unknown | ACGTTGGATGACCTACTGGACTCTATTGTG  ACGTTGGATGCCCCTTGACTCACATAATGG | TGTAATTTGCTTCATAGTTTAGACAC |
| 12654933 | C/A | 110430654 | Unknown | ACGTTGGATGGCTTTATGCCTTGGAAGCAG  ACGTTGGATGAGGTGAACTCAACCAGAAGC | GGAAGCAGACCCATAAG |
| 10455025 | A/C | 110432898 | Unknown | ACGTTGGATGAGGAGACCACTTTTGGAGAC  ACGTTGGATGCTCTGAGCTGTTTTCTTCCC | CAAATAAGATGAACATGGCA |
| 11466741 | C/T | 110436604 | nearGene-5 | ACGTTGGATGCATTTTGGAGAGGGAGTATC  ACGTTGGATGAGCCCAGATGTCCCCTTTC | GGAGAGGGAGTATCCTGCTA |
| 13156086 | A/C | 110443368 | Unknown | ACGTTGGATGCTATTGCCACAAAGCAGTCC  ACGTTGGATGGGAATAAAGACTGTTACAC | AGACAGAAAACTAAAAATAAAGTTAC |
| 6886755 | G/T | 110443500 | Unknown | ACGTTGGATGGCACACAGTTATTGACAGCC  ACGTTGGATGTTCTCTGTGCACCTCTTACC | TAGCAGAGTTCATAAGACA |
| 252706 | G/A | 110444759 | Unknown | ACGTTGGATGGATTTCTACCTGTTAGCTTC  ACGTTGGATGTCAAGAATAGTGGAGAATGG | CTGTTTAAGTAGGAGGGT |
| 2416259 | T/C | 110447641 | Unknown | ACGTTGGATGGTCATCCTTGGACACACAAC  ACGTTGGATGGCCACAGGTTCACAAAGGAC | gTTGGAAACCTTAACATCATATT |

JAI GANPATI BAPA JAI CHAMUNDA MATA JAI HANUMAN DADA JAI JALARAM BAPA
